# Smile dimensions affect self-perceived smile attractiveness

**DOI:** 10.1038/s41598-021-82478-9

**Published:** 2021-02-02

**Authors:** Simone Horn, Natalia Matuszewska, Nikolaos Gkantidis, Carlalberta Verna, Georgios Kanavakis

**Affiliations:** 1 Department of Pediatric Oral Health and Orthodontics, University Center for Dental Medicine (UZB) / University of Basel, Basel, 4058 Switzerland; 2 Department of Orthodontics and Dentofacial Orthopedics, University of Bern, Bern, 3010 Switzerland; 3 Department of Orthodontics, Tufts University School of Dental Medicine, Boston, 02111 MA USA

**Keywords:** Orthodontics, Human behaviour, Dentistry, Medical imaging

## Abstract

Facial expressions play a leading role in human interactions because they provide signaling information of emotion and create social perceptions of an individuals’ physical and personality traits. Smiling increases socially perceived attractiveness and is considered a signal of trustworthiness and intelligence. Despite the ample information regarding the social importance of an attractive smile, little is known about the association between smile characteristics and self-assessed smile attractiveness. Here we investigate the effect of smile dimensions on ratings of self-perceived smile attractiveness, in a group of 613 young adults using 3D facial imaging. We show a significant effect of proportional smile width (ratio of smile width to facial width) on self-perceived smile attractiveness. In fact, for every 10% increase in proportional smile width, self-perceived attractiveness ratings increased by 10.26%. In the present sample, this association was primarily evident in females. Our results indicate that objective characteristics of the smile influence self-perception of smile attractiveness. The increased strength of the effect in females provides support to the notion that females are overall more aware of their smile and the impact it has on their public image.

## Introduction

The social impact of facial appearance and expression is well documented; more attractive individuals are viewed preferably in their social interactions and tend to be more successful in most aspects of life that modern society considers to be important^1,2^. There are various facial features that contribute to describing a face as attractive, including overall shape^3,4^, symmetry^5^, averageness^2^ and others. However facial expression tends to also play an important role. Using functional magnetic resonance imaging (f-MRI), a stronger neuronal response in the medial orbitofrontal cortex was observed, when observing a smiling face compared to a face with neutral expression^6^. Facial expressions of alertness and positive mood are indicators of intelligence^7^, and facial expression of happiness strongly contributes to the development of personal biases when humans make visual contact; thus, smiling tends to play a primary role in our daily social interactions, more so than other physical traits^8^. In addition, smiling creates a social perception of happiness, youthfulness and kindness^9–11^ and certain features of an attractive smile have also been correlated to various personality traits, such as neuroticism, self-esteem and dominance^12^.

An improvement in smile esthetics is also the main reason for patients to seek various treatments of the perioral region^13–15^. An excessive gingival display upon smiling, namely a “gummy smile”, for instance, is often associated with hypermobility of the upper lip^13^, which is then often managed with the use of botulinum toxin injections^14^. Furthermore, dissatisfaction with smile attractiveness is the leading motivational factor for adult patients to seek orthodontic care^15^.

The attractiveness of a smile has been related to the thickness and the position of the upper lip, the amount of tooth exposure, the presence of black triangles and occlusal cants, as well as the extent of gingival display^12,16,17^. However, the dimensional characteristics of an attractive smile have not been studied yet, with the exception of interlabial height, which has been found to be larger in smiles that were judged as “attractive” by external evaluators^18^. In addition, there is scarce information regarding self-perceived smile attractiveness despite the significant impact it has on patients’ treatment goals and clinicians’ treatment strategies.

Moreover, previous assessments of smile attractiveness have mostly used 2D photographs, which by definition include various sources of error, namely, lens distortion and magnification^19^, patient positioning^20,21^ and landmark identification. Three-dimensional surface imaging (stereophotogrammetry), on the other hand, is currently the most reliable and advantageous method for dimensional assessment of facial soft tissues^22–24^. Inaccuracies in stereophotogrammetry may stem from the technical error related to the camera system, involuntary subject movement and landmark identification error^23–26^. The latter has been found to be less than 0.2 mm for most facial landmarks and never more than 1 mm^24,25^. The 3D camera error is considered negligible, and significantly less than the error resulting from involuntary subject movement that should preferably be controlled in order to avoid measurement discrepancies^26^.

Based on the above considerations, the aim of this study was to determine the dimensional characteristics of an attractive smile, using self-assessment tools and three-dimensional surface imaging. More specifically, our hypothesis was that smile width and smile height affect self-perceived smile attractiveness.

## Materials and methods

### Ethical approval

This study is part of a larger exploratory investigation that was reviewed and approved by the Health Sciences Institutional Review Board (IRB) of Tufts University in Boston, Massachusetts (IRB#: 11181). The methodology was carried out in accordance with the relevant guidelines and regulations and all participants signed an informed consent before any study procedures were performed.

### Study sample

The study population consisted of 613 (214 males; 399 females) volunteers, all pre-doctoral students at Tufts University, Health Sciences campus in Boston MA. All participants were young adults, aged 21–35 years, who spoke English as their native language, and did not have any craniofacial symptoms, visible deformations of the face or a history of facial reconstructive surgery.

### Methodology

**Figure 1:**
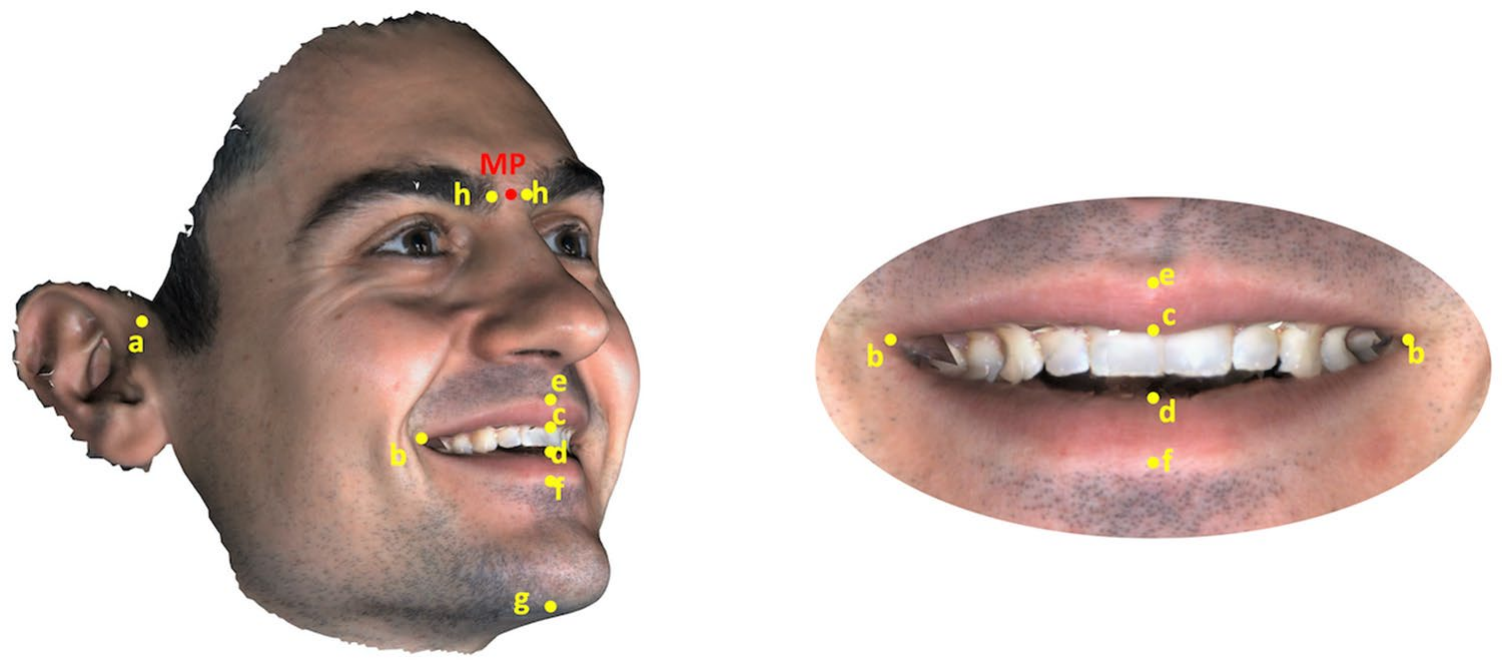
Selected landmarks on 3D images: (**a**) Right and left most lateral facial point (at the intersection where the outer outline of the ear connects to the face); (**b**) Right and left corner of the mouth; (**c**) Upper stomion (the midpoint of the upper lip inferior border); (**d**) Lower stomion (the midpoint of the lower lip superior border); (**e**) Upper vermillion midpoint (the midpoint of the upper lip superior border); (**f**) Lower vermillion midpoint (the midpoint of the lower lip inferior border); (**g**) Menton (the most inferior point of the median plane of the chin); (**h**) Right and left most median point of the upper eyebrow ridge. (Image was created using Viewbox 4 software (version 4.1.0.1 BETA, http://www.dhal.com/viewboxindex.htm). The projection of the midpoint between the right and left most median points of the upper eyebrow ridge on the surface image was considered the most superior midline point of the face (point MP) and was used to define facial height. (Image was created using Viewbox 4 software (version 4.1.0.1 BETA, http://www.dhal.com/viewboxindex.htm).

Smiling images were captured on a single visit using a 3D stereophotogrammetry system (3DMD, Atlanta, Georgia). According to the guidelines of the camera system manufacturer, participants were seated on a chair positioned at a standardized distance (approximately 70 cm) from the camera, with their head slightly tilted upwards (c.a. 10 degrees to the horizontal plane). They were asked to perform a social smile (smiling photo). All images were acquired by one experienced person (G.K.) for standardization purposes. Three-dimensional images were uploaded on “VIEWBOX 4.1” software (dHal Software, Kifissia, Greece) and digitized. The selected landmarks for digitization are depicted and described in Fig. 1 and were used to measure smile width and height, proportional smile width and height, upper vermillion height and lower vermillion height (Table 1). All digitizations were performed by two calibrated operators, who were able to move and scale the images as preferred, to facilitate good landmark identification. Each operator executed the digitization process twice for 40 randomly selected images to test inter- and intra-operator agreement.

**Table 1:** Description of measured smile dimensions.

Smile dimensions	Description
Smile width	Distance between the right and left corner of the mouth
Smile height	Distance between upper and lower stomion
Face width	Distance between the right and left most lateral facial points
Face height	Distance between Menton and Point MP
Upper vermillion height	Distance between upper vermillion midpoint and upper stomion
Lower vermillion height	Distance between lower vermillion midpoint and lower stomion
Proportional smile width	Ratio of smile width to facial width
Proportional smile height	Ratio of smile height to facial height

In addition to posing for a 3D smiling image, each individual was asked to complete a questionnaire with basic demographic information and then perform a self-assessment of their smile attractiveness by manually placing a mark on a simple 100 mm visual analogue scale (VAS) scale^27^. The question asked was: “How would you rate the esthetic appearance of your smile?”, with possible scores ranging between “completely unattractive” and “extremely” attractive. Participants were instructed to place a vertical mark on the VAS scale to indicate their answer. Questionnaires were completed privately. However, a research coordinator was present in the room to answer any logistic questions.

A research team member, who was not involved in any other part of the study and was not informed about its precise aim, was assigned to measure the distance between the starting point of the VAS scale (“completely unattractive”) and the vertical marks; and record the measurements (to the second decimal digit) in an Excel sheet (Microsoft Excel, Redmond WA, USA), using a digital caliper. These measurements indicated participants’ answers to the study question and were considered as the primary outcome. In order to calculate error from data insertion, fifty randomly selected questionnaires were reviewed twice by the same operator and the entire process of measuring and inserting measurement values was repeated at least one month after the first time.

### Statistical analyses

A regression model was developed to assess the effect of sex, age and smile dimensions on self-perceived smile attractiveness (dependent variable). In addition, due to the various inherent biological differences between sexes, including facial dimensions and morphology^1^, it was considered to also study the main hypothesis separately in males and females. A Student’s t-test for independent samples was conducted to explore sexual differences in demographic information, self-perceived attractiveness scores, and smile dimensions and confirmed the presence of significant differences between sexes (Table 2). Therefore, all following regression analyses were performed separately in males and females.

**Table 2:** Differences between males and females in age, self-perceived smile attractiveness scores and smile dimensions.

Variable	Sex (N)	Mean	SD	*p* value
Age (years)	Male (214)	25.79	2.50	0.010
	Female (399)	25.27	2.12	
Self-perceived smile attractiveness	Male (214)	67.54	17.31	0.015
	Female (399)	70.94	15.94	
Smile width	Male (214)	63.90	4.99	0.007
	Female (399)	62.80	4.66	
Smile height	Male (214)	10.71	2.75	0.315
	Female (399)	10.94	2.63	
Face width	Male (214)	155.42	8.87	0.359
	Female (399)	154.78	7.81	
Face height	Male (214)	134.38	8.57	0.450
	Female (399)	134.92	8.43	
Proportional smile width	Male (214)	0.398	0.033	0.001
	Female (399)	0.414	0.036	
Proportional smile height	Male (214)	0.079	0.017	0.083
	Female (399)	0.081	0.020	
Upper vermillion width	Male (214)	7.53	1.67	0.987
	Female (399)	7.53	1.56	
Lower vermillion width	Male (214)	8.78	1.68	0.001
	Female (399)	8.32	1.57	

In order to explore the effect of smile dimensions on self-perceived smile attractiveness, a multiple linear regression model was applied, with “self-perceived smile attractiveness” as the dependent variable. The predictor variables used in the model were: age, smile width, smile height, upper vermillion height, lower vermillion height, proportional smile width and proportional smile height. Prior to running the regression model, the assumption of normality was tested for each subsample with P-P plots of the regression residuals (Supplemental Fig. 1), which displayed a normal distribution in both cases. The presence of homoscedasticity within each sample (males/females) was assessed by plotting the standardized residuals against the predicted values (Supplemental Fig. 2); the scatterplots showed that the assumption of homoscedasticity was met in both cases. In order to explore the presence of multicollinearity between the predictive variables of the regression models, the variance inflation factors (VIF) and individual correlation coefficients were evaluated. The only high correlation coefficients detected were between “smile width” and “proportional smile width”, which was an expected finding due to their obvious relevance. However, for both variables, the variance VIF was within acceptable limits (< 5), and because both variables were considered important predictive factors for our primary research question, they were both included in the final regression model. All statistical analyses were performed with SPSS version 26.0 (IBM, Armonk, NY) and VIEWBOX 4 software, version 4.1.0.1 BETA (dHal Software, Kifissia, Greece). The level of statistical significance was set at *p* = 0.05.

**Figure 2:**
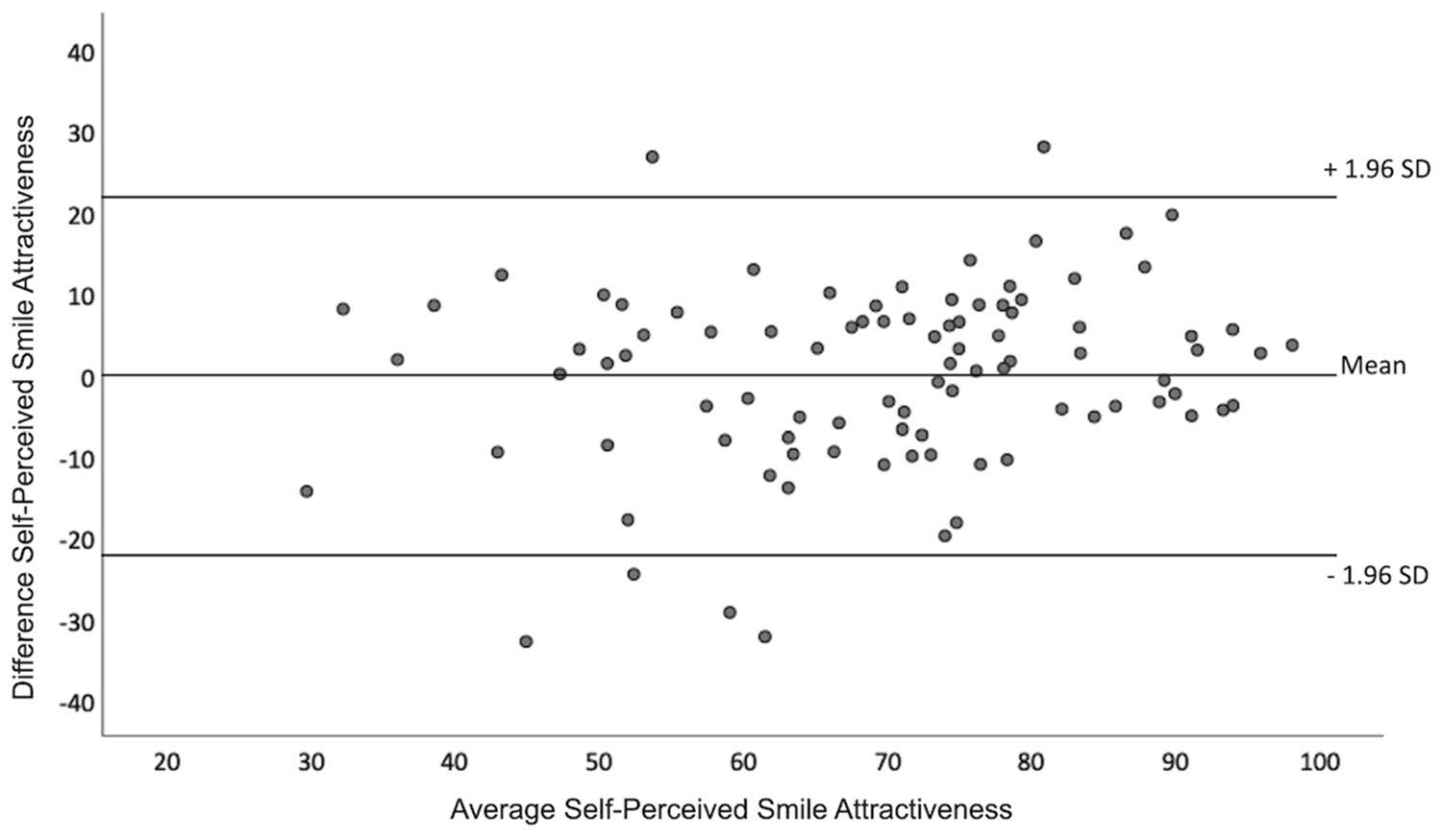
Bland–Altman plot showing the level of agreement in self-perceived facial attractiveness between the first and second visits of 93 subjects. (Graph was created using SPSS version 26.0, https://www.ibm.com/products/spss-statistics).

## Results

### Error assessment

In order to assess the error of the questionnaire section, 93 random subjects who agreed to return for a follow-up visit were photographed again and were asked to complete the questionnaire for a second time. A two-sided t-test for independent samples showed no systematic error between two visits (*p* = 0.971). Also, based on the distribution of points in the Bland–Altman plot, there was a high agreement in the subjects’ answers between the first and second visits, which is indicative of acceptable random error in our primary outcome (Fig. 2).

Digitization error was assessed as intra- and inter-rater agreement using the linear and proportional variables included in our regression model. Both, intra- and inter-rater assessments did not present any systematic error (*p* > 0.05), and random error was acceptable in both instances (Supplementary Tables 1 and 2).

The measurement error during recording of VAS scales onto an Excel worksheet was explored with Bland–Altman plots and was found to be minimal (Mean difference in VAS measurements: 0.3112; Lower 95% limit of agreement: − 0.593; Upper 95% limit of agreement: 1.215).

### Self-perceived smile attractiveness

The regression model showed a significant effect of the proportional smile width on self-perceived smile attractiveness (Unstandardized B = 102.64; *p* = 0.005), while sex, age and all other examined factors were not significantly associated to the primary outcome (Table 3). For every 10% increase in proportional smile width, self-perceived attractiveness increased by 10.26 points on the VAS scale. Although sex did not appear to affect self-perceived smile attractiveness, the research question was also assessed separately in males and females due to the well-established biological differences between sexes and because their facial dimensions were evidently different in the present sample (Table 2).

**Table 3:** Regression of self-perceived attractiveness on sex, age and smile dimensions.

Dependent variable	Parameter	β-coefficient	95% CI		
			Lower bound	Upper bound	*p* value
Self-perceived attractiveness	Intercept	32.659	8.147	57.172	0.009
	Age	− 0.203	− 0.769	0.363	0.482
	Male (female: reference)	− 1.778	− 5.000	1.444	0.279
	Smile width	− 0.010	− 0.536	0.516	0.970
	Smile height	0.310	− 0.178	0.798	0.212
	Upper vermillion height	− 0.464	− 1.439	0.511	0.351
	Lower vermillion height	− 0.418	− 0.543	1.379	0.393
	Proportional smile width	102.646	30.407	174.885	0.005
	Proportional smile height	− 23.056	− 90.015	43.903	0.499

**Figure 3:**
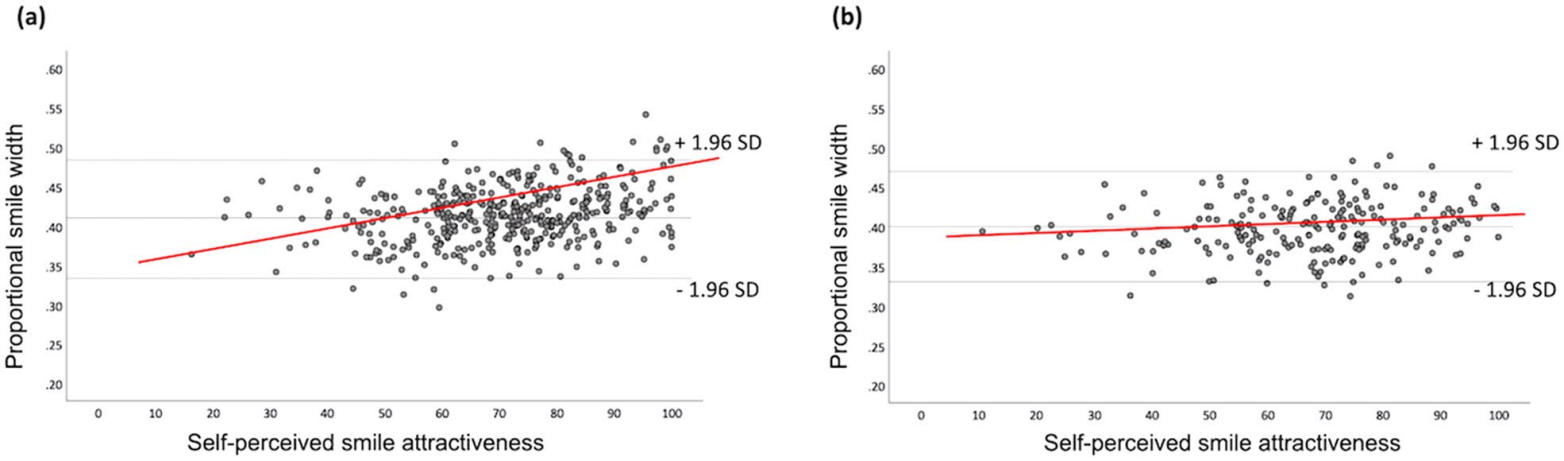
Linear regression of proportional smile width on self-perceived smile attractiveness, in (**a**) females and (**b**) males. (Images were created using SPSS version 26.0, https://www.ibm.com/products/spss-statistics).

The regression analysis in females showed that self-perceived smile attractiveness presented a significant association to smile dimensions (*p* < 0.001). The smile variables used in the model predicted 7.7% (Adjusted R^2^ = 0.077) of the variability in self-perceived smile attractiveness, indicating that participants’ answers were also influenced by factors other than smile dimensions. The only variable, again, that appeared to have a significant effect on the primary outcome was proportional smile width (Table 4; Fig. 3). More specifically, for every 10% increase in proportional smile width in females, there was an increase of 11.7 in smile attractiveness scores on the VAS (Standardized β = 0.265; *p* = 0.005). In males, the regression model presented a non-significant association to the primary outcome and negligible predictive value (Adjusted R^2^ = −0.008; *p* = 0.612), indicating that none of the studied smile dimensions had an effect on self-perceived smile attractiveness scores (Table 4; Fig. 3).

**Table 4:** Regression coefficients of predictor variables, displaying their individual effect on self-perceived smile attractiveness in females and males.

	Multiple linear regression		
	Predictors	Coefficients	
		Standardized coefficients beta	*p* value
Females	Age	− 0.070	0.150
	Smile width	− 0.007	0.939
	Smile height	0.079	0.113
	Upper vermillion height	− 0.056	0.356
	Lower vermillion height	0.067	0.267
	Proportional smile width	0.265	0.005
	Proportional smile height	− 0.009	0.846
Males	Age	0.040	0.567
	Smile width	0.021	0.885
	Smile height	− 0.003	0.960
	Upper vermillion height	− 0.043	0.603
	Lower vermillion height	0.015	0.850
	Proportional smile width	0.121	0.401
	Proportional smile height	− 0.069	0.326

## Discussion

The purpose of this study was to explore the effect of smile dimensions on self-assessment of smile attractiveness in a large young adult population, using three-dimensional data. To our knowledge this is the first assessment of the association of self-perceived smile attractiveness to smile dimensions. The findings demonstrated that self-perceived smile attractiveness was affected by smile dimensions, with proportionally wider smiles perceived as more attractive. This effect was primarily evident in females.

As the societal esthetic demands influence interpersonal relationships heavily, medical and dental disciplines studying the human face continue to shift their focus on treatment trajectories that optimize facial and smile esthetics. Following an intervention in the face, self-perceived esthetic outcomes comprise important components of patient satisfaction with treatment^28,29^. Smiling can trigger a variety of emotions and biases during human interactions^8,10,11^ and it may be the most important factor controlling judgments of overall facial attractiveness^30^. Thus, the identification of factors that affect self-perceived smile attractiveness is important to set treatment goals that meet patients’ needs and demands, when treatment is expected to affect smile.

We were able to identify only one previous study associating smile characteristics to self-perceived smile attractiveness^12^. This was conducted on a sample of white adult men, who were asked to evaluate their smiles according to tooth size and color, tooth visibility, and upper lip position, while viewing their smiling image. The results indicated that tooth size and color appeared to have a larger effect on attractiveness ratings, however, smile dimensions were not evaluated and therefore no direct comparisons to our results are possible.

Considering external ratings, in growing individuals, a thicker upper lip was shown to influence observers’ judgements of an attractive smile^31^; while another study on young adult females reported that only smile height was related to smile attractiveness^18^. On the other hand, Schabel et al.^32^ were not able to identify any dimensional smile characteristic with a direct effect on smile attractiveness. Apart from the external ratings, the above studies had samples much smaller to ours (48–60 subjects), tested 2D images, and had different designs. Thus, no direct comparison to our study findings can be made.

No previous study has assessed smile dimensions in relation to face dimensions (proportional smile dimensions). During social interactions, attention is primarily shifted between the mouth and the eyes^33^; and thus, in real life, dimensional characteristics of facial structures are mostly viewed in relation to others. Therefore, it is safe to assume that quantitative assessments, which take the variability in facial size and shape into consideration are more likely to provide realistic information about smile dimensions. Here, we show that smile width, as related to facial width, has a significant effect on self-perceived smile attractiveness; participants with proportionally wider smiles, found their smiles more attractive. No such effect was evident for the original smile width.

Our sample population exhibited significant sexual dimorphism in smile dimensions, with males presenting wider smiles and faces than females. This is in agreement with previous studies that have explored smile dimensions using three-dimensional data^34–37^. However, it can be attributed to males exhibiting overall larger facial dimensions, since in our sample females had higher proportional smile widths. Our findings also allow for speculation that young adult women are more influenced by their objective smile appearance when evaluating their own smile attractiveness. Although smiling kindles an equally favorable response in females and males during social encounters^6^, females are consistently found to smile more^38^. This might be an inherent sexual characteristic of females or they may pay more attention or even be more aware of the positive responses generated during smile, and thus, exert a more conscious effort to smile. The latter might be supported by our finding that in contrast to young adult females, in males, smile dimensions had an undetectable effect on self-perceived smile attractiveness. There are numerous social and cultural causes for women being more conscious of their objective smile characteristics, since they are often expected to be friendlier and more emotionally expressive than men^39–41^. In addition, smiling frequency and intensity has been associated to hormonal changes during physical development; high testosterone levels, for example, have an inhibitory role in social smiling^42,43^. As a result, males may either have an intrinsic hesitation to smile or may very well not consider it an important feature of their social image. This could potentially lead them to rate their smile less favorably compared to females, which was clearly evident from our results and, thus, also supports the above thought process.

### Special considerations and limitations

The results of this investigation should be assessed within the context of the applied methodology. Our study population was limited to a group of highly educated young adults, in order to control for the potential confounding effect of educational status^44,45^ and age^46^. The findings may thus not represent the general population.

In addition, participants were not allowed to look at their own pictures prior to evaluating their attractiveness. Being exposed to one’s own photograph tends to alter self-perception of appearance^47^, therefore this could have led to different results. However, it was preferred to obtain a more “genuine” response in order to avoid the effect of the instantaneous stimulus generated from the exposure to their facial images. In addition, some participants may have been influenced when smiling by the presence of a person in the research area to whom they wanted to appear attractive. Although this could potentially affect our results, it is unlikely given the large sample size and the minimal measurement error found.

The present study focused solely on smile dimensions. However, there are various other smile components that might affect smile attractiveness, but they were not considered in our study. Such factors could have been expected to confound ratings and affect the study outcomes, but we think that the large sample size adequately addresses this issue.

Anatomical landmark variability could also be a possible source of error in this study since the labial commissures of the mouth tend to not displace consistently upon smiling, between different image acquisitions of the same individual^48^. However, this confounder is not expected to have influenced our results due to the large sample population and the high standardization of image acquisition.

## Conclusion

In young adults, the width of the smile as related to facial width is an important factor affecting their self-assessment of smile attractiveness. Although sex did not appear to influence this association, in the present sample population, the effect of smile dimensions primarily discernible in females. This finding delivers important diagnostic information to orthodontics, orthognathic surgeons and other clinicians who specialize in improving the facial and smile esthetics.

## Supplementary Information

Supplementary Information.
