# Supplementary material for: Smile dimensions affect self-perceived smile attractiveness
Source: Sci Rep. 2021 Feb 2;11:2779. doi: 10.1038/s41598-021-82478-9 (PMC7854600; doi:10.1038/s41598-021-82478-9)
Supplement: Supplementary file 1 — Supplementary Information. [file 41598_2021_82478_MOESM1_ESM.docx]

**Supplementary material**

**Smile dimensions affect self-perceived smile attractiveness**

Simone Horn^1*^, Natalia Matuszewska^1*^, Nikolaos Gkantidis^2^, Carlalberta Verna^1^, Georgios Kanavakis^1,3^

^1^Department of Pediatric Oral Health and Orthodontics, University Center for Dental Medicine (UZB), University of Basel, Switzerland

^2^Department of Orthodontics and Dentofacial Orthopedics, University of Bern, Switzerland

^3^Department of Orthodontics, Tufts University School of Dental Medicine, Boston MA, USA

*The first two authors contributed equally to this manuscript

**Short Title:** Smile dimensions and self-perceived smile attractiveness

**Keywords:** Self-perceived smile attractiveness, stereophotogrammetry, surface imaging, smile attractiveness, smile dimensions

**Corresponding author:**

Georgios Kanavakis, Department of Pediatric Oral Health and Orthodontics

University Center for Dental Medicine - UZB

Mattenstrasse 40

CH-4058 Basel

Tel: +41 (0) 61 267 26 15

Fax: +41 (0) 61 267 25 81

Email: georgios.kanavakis@unibas.ch


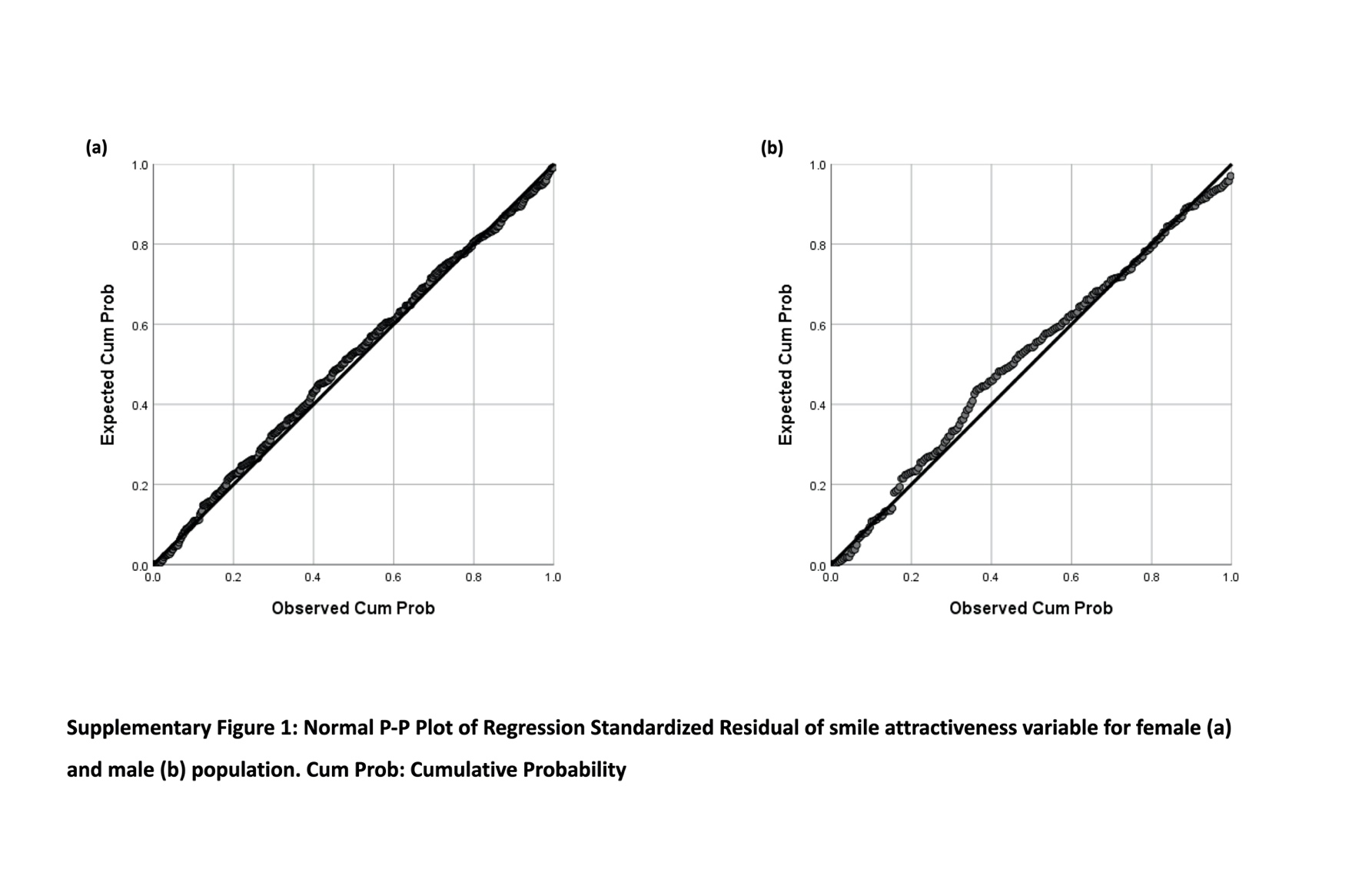


Supplementary Figure 1: Normal P-P Plot of Regression Standardized Residual of smile attractiveness variable for female (a) and male (b) population. Cum Prob: Cumulative Probability


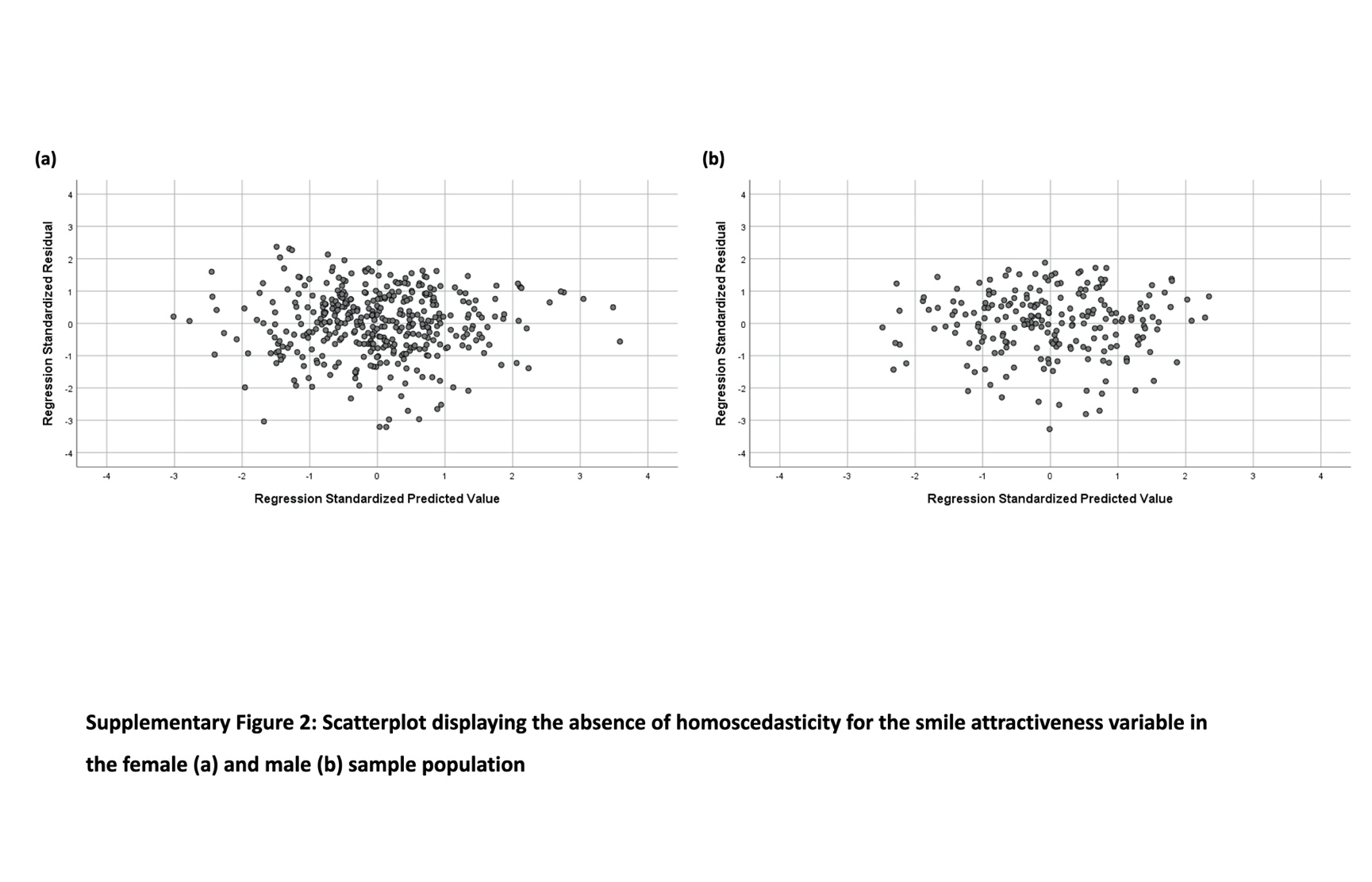


Supplementary Figure 2: Scatterplot displaying the absence of homoscedasticity for the smile attractiveness variable in female (a) and male (b) sample population.

Supplementary Table 1: Inter-rater error assessment

1. Two-tailed t-test displaying the absence of systematic error between the two raters

| Variable | Rater | Mean | SD | *P*-value |
| --- | --- | --- | --- | --- |
| Smile width | 1 | 60.700 | 5.512 | 0.646 |
|  | 2 | 61.664 | 5.474 |  |
| Smile height | 1 | 10.486 | 3.157 | 0.913 |
|  | 2 | 10.357 | 2.982 |  |
| Upper vermillion height | 1 | 7.936 | 2.053 | 0.911 |
|  | 2 | 7.850 | 1.980 |  |
| Lower vermillion height | 1 | 8.779 | 1.953 | 0.187 |
|  | 2 | 9.786 | 1.982 |  |
| Proportional smile width | 1 | 0.377 | 0.055 | 0.717 |
|  | 2 | 0.385 | 0.057 |  |
| Proportional smile height | 1 | 0.073 | 0.021 | 0.944 |
|  | 2 | 0.072 | 0.020 |  |

1. Mean differences and limits of agreement displaying acceptable random error between raters

| Variable | Mean difference (Δ) | Limits of agreement | |
| --- | --- | --- | --- |
|  |  | Upper (+1.96 SD) | Lower (-1.96 SD) |
| Smile width | -0.964 | 0.733 | - 2.662 |
| Smile height | 0.129 | 0.979 | -0.722 |
| Upper vermillion height | 0.086 | 1.845 | -1.671 |
| Lower vermillion height | -1.0071 | 0.496 | -2.510 |
| Proportional smile width | -0.008 | 0.011 | -0.025 |
| Proportional smile height | 0.001 | 0.006 | -0.005 |

Supplementary Table 2: Intra-rater error assessment

| Variable | Rating | Mean | SD | *P*-value |
| --- | --- | --- | --- | --- |
| Smile width | 1 | 60.807 | 5.297 | 0.992 |
|  | 2 | 60.820 | 5.338 |  |
| Smile height | 1 | 11.285 | 3.157 | 0.597 |
|  | 2 | 10.915 | 3.067 |  |
| Upper vermillion height | 1 | 8.492 | 1.728 | 0.596 |
|  | 2 | 8.690 | 1.591 |  |
| Lower vermillion height | 1 | 8.897 | 1.799 | 0.255 |
|  | 2 | 9.337 | 1.631 |  |
| Proportional smile width | 1 | 0.380 | 0.044 | 0.922 |
|  | 2 | 0.381 | 0.044 |  |
| Proportional smile height | 1 | 0.080 | 0.021 | 0.607 |
|  | 2 | 0.078 | 0.020 |  |

1. Two-tailed t-test displaying the absence of systematic error between the two ratings
2. Mean differences and limits of agreement displaying acceptable random error between ratings

| Variable | Mean difference (Δ) | Limits of agreement | |
| --- | --- | --- | --- |
|  |  | Upper (+1.96 SD) | Lower (-1.96 SD) |
| Smile width | -0.012 | 2.154 | -2.178 |
| Smile height | 0.370 | 1.174 | -0.434 |
| Upper vermillion height | -0.197 | 0.938 | -1.332 |
| Lower vermillion height | -0.440 | 0.530 | -1.410 |
| Proportional smile width | -0.001 | 0.015 | -0.017 |
| Proportional smile height | 0.002 | 0.008 | -0.004 |
